# The Impact of Aggressive Conditions on the Mechanical and Rheological Properties of Components Produced Using Additive Manufacturing

**DOI:** 10.3390/ma18214917

**Published:** 2025-10-28

**Authors:** Iwona Michalska-Pożoga, Katarzyna Bryll, Radosław Patyk, Marcin Szczepanek

**Affiliations:** 1 Faculty of Mechanical and Energy Engineering, Koszalin University of Technology, ul. Raclawicka 15-17, 75-620 Koszalin, Poland; radoslaw.patyk@tu.koszalin.pl; 2 Faculty of Marine Engineering, Maritime University of Szczecin, ul. Waly Chrobrego 1-2, 70-500 Szczecin, Poland; k.bryll@pm.szczecin.pl (K.B.); m.szczepanek@pm.szczecin.pl (M.S.)

**Keywords:** polymer-wood composite (PLA + MD), recycled polylactide (rPLA), Fused Deposition Modeling (FDM) additive manufacturing technologies, aging processes, seawater

## Abstract

Analysis of the impact of aging processes induced by environmental conditions, particularly aggressive ones, on the properties of polymeric materials and products made from them has been the subject of intensive research for many years. Developing materials characterized by high resistance to the specific external factors in which these materials are used is a key issue in the context of developing a sustainable economy aimed at minimizing waste and extending the service life of polymeric components. The main objective of this research was to assess and quantify the degradation mechanisms of polymeric materials manufactured using additive Fused Deposition Modeling (FDM) technology when exposed to aggressive marine environments. To achieve this, the study analyzed the influence of seawater corrosion conditions on the changes in mechanical and rheological properties of two polymeric materials: recycled polylactide (rPLA) and a wood–polymer composite (WPC) based on PLA reinforced with wood flour (MD). The results revealed that rPLA exhibited an approximately 16% decrease in average molecular weight after 9 months of seawater exposure, accompanied by a 37% reduction in tensile strength and a 24% decrease in elastic modulus. In the case of the WPC, the molecular weight decreased by about 20%, while tensile strength and elastic modulus dropped by 30% and 51%, respectively. The findings provide quantitative evidence of the susceptibility of additively manufactured biodegradable polymers to marine-induced degradation, highlighting the necessity of further optimization for maritime and coastal applications.

## 1. Introduction

Currently, polymer composites, particularly those with natural additives, are experiencing increasing widespread use due to their favorable static and physical properties and resistance to environmental conditions, especially in aggressive environments, which allows for high corrosion resistance. Compared to traditional materials such as metals, they are characterized by low weight and high mechanical strength. Implementing polymer composites in marine environments is currently a key technological challenge. These materials are commonly used in the construction of hulls for vessels such as boats, yachts, cutters, and small warships. They are also used in the production of accessories such as masts, deck coverings, furniture components, and structural components for drilling platforms [1,2,3,4,5,6,7,8,9].

Exposing polymeric products to external factors that cause changes in their physical and chemical properties may result in a loss of functional properties and deterioration of their parameters, such as increased brittleness, the appearance of microcracks, color change, and surface roughening. These processes are referred to as aging of polymeric materials and are irreversible. Key chemical reactions occurring during aging include depolymerization, destruction, degradation (chemical, thermal, biological, mechanical, and photochemical), as well as cross-linking and oxidation (thermo-oxidation) [10,11,12,13,14,15,16,17,18,19]. Factors influencing the aging processes of polymers include: oxidants, such as oxygen and ozone; increased temperature and its cyclic, rapid changes; the impact of electromagnetic radiation in the UV range and radiation; aggressive chemicals, including water, water vapor, and marine environments; and mechanical stresses, with particular emphasis on cyclic dynamic stresses. The factors inducing polymer aging presented here demonstrate varying effectiveness depending on the type of polymer material being analyzed. The rate of thermal, mechanical, and chemical degradation of polymer materials is primarily determined by their physicochemical properties, such as molecular structure, molecular weight, crystallinity, the presence of additives, and the degree of cross-linking. Factors accelerating polymer aging do not act individually but usually synergistically, resulting in complex and mutually reinforcing degradation mechanisms [12,15,16,20,21,22].

Polymer degradation in the marine environment occurs through various chemical and biological processes. The extent of these processes is influenced by the material’s physicochemical properties and environmental factors, including temperature, salinity, solar radiation intensity, and the presence of microorganisms [23,24]. Polylactide (PLA), an aliphatic polyester, degrades primarily through hydrolysis of ester bonds, which leads to the gradual disintegration of polymer chains and a reduction in molecular weight. This process occurs more rapidly at elevated temperatures, meaning that in the cool waters of the Baltic Sea, its rate is limited but still noticeable [25]. Hydrolysis leads to the formation of shorter oligomers and monomers, which can be metabolized by bacteria. As hydrolysis progresses, the importance of enzymatic biodegradation increases, catalyzed by microorganisms that produce esterases and lipases, capable of further degrading polymer fragments into lactic acid, which can then be used in the microbial metabolic processes [26]. Photodegradation of PLA under the influence of UV radiation can initiate oxidative reactions, accelerating surface degradation [27].

Wood–polymer Composite contains both a polymer fraction and an organic component, i.e., wood flour. Degradation of this material occurs through mechanisms characteristic of both components. Degradation processes occurring in the polymer matrix are similar to those observed in pure polymers, while the presence of wood flour significantly accelerates composite degradation, acting as a source of substrate for microorganisms. Microorganisms colonizing the material’s surface increase its porosity, facilitating water penetration and access of biodegradable enzymes to the deeper structure [28]. Furthermore, lignocellulosic components present in the composite undergo biodegradation under the influence of cellulolytic enzymes, leading to gradual fragmentation and structural disintegration of the material.

Modern materials used in innovative technologies in various sectors should be characterized by high efficiency and effectiveness, as well as ensure operational reliability. Furthermore, they must be lightweight, highly resistant to extreme environmental conditions, such as extreme temperatures (high and low), high and low pressures, as well as resistant to aggressive substances and unfavorable environmental conditions. One of the most popular additive manufacturing techniques currently is Fused Deposition Modeling (FDM). FDM is a method involving the layer-by-layer deposition of molten material. FDM is becoming an increasingly popular manufacturing technology due to its low production and maintenance costs and the growing variety of materials used, such as polylactic acid, polypropylene (PP), polyethylene terephthalate glycol (PETG), and acrylonitrile butadiene styrene (ABS) [29,30].

The main objective of the present research was to investigate and quantify the effects of long-term exposure to aggressive marine environments on the degradation mechanisms and mechanical performance of additively manufactured polymeric materials produced by FDM technology. The study focused on two representative biodegradable materials—recycled PLA (rPLA) and a wood–polymer composite (WPC) based on PLA with wood flour reinforcement—aiming to evaluate their suitability and long-term stability for potential maritime and coastal applications.

## 2. Materials and Methods

### 2.1. Research Material

**Figure 1:**
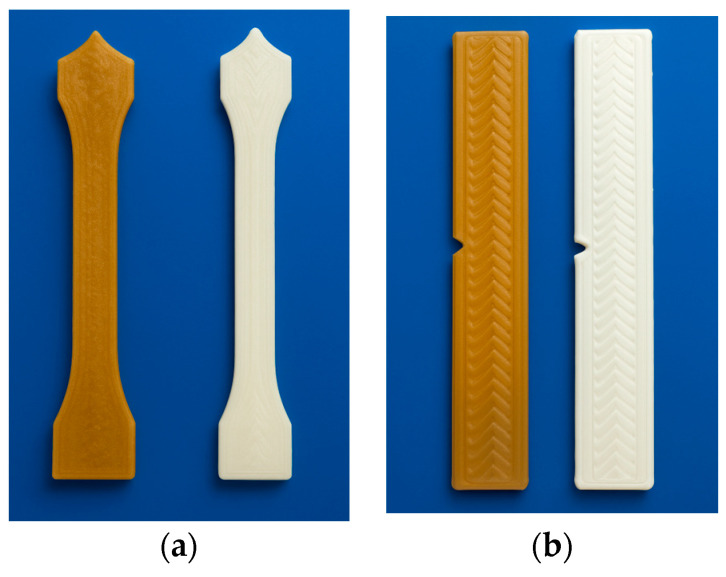
Test specimen made in FDM additive technology: (**a**) test specimen for mechanical properties under static tension, (**b**) test specimen for Charpy impact strength, in order from the left: WPC (PLA + MD), rPLA.

The research material consisted of shapes manufactured from commercial filaments using FDM (Fused Deposition Modeling) additive technology. Two types of specimens were prepared: a test specimen for mechanical properties under static tension (Figure 1a) and a test specimen for Charpy impact strength (Figure 1b). The materials used were a biopolymer—recycled polylactide (rPLA) from Spectrum, Poland, and a WPC polymer composite based on polylactide with the addition of wood flour (PLA + MD), marketed under the trade name WOOD and also produced by Spectrum, Poland. The specimens were fabricated using a Creality Ender-3 printer equipped with a direct-drive extruder, ensuring stable filament feeding and uniform material deposition during the printing process.

The filament properties have been presented in Table 1, and Additive manufacturing processes using FDM technology were carried out with the 112 parameters shown in Table 2.

### 2.2. Research Methods

The research material was subjected to a degradation process using natural seawater from the Baltic Sea, characterized by the following parameters: pH = 7.9 and conductivity = 2978 μS cm^−1^. The samples were placed in sealed tanks, ensuring a constant temperature of 23 ± 2 °C and periodic monthly seawater exchange to maintain stable chemical conditions. The samples were stored for nine months. The tests were conducted in a three-month sequence, i.e., at months 0, 3, 6, and 9.

The research material was subjected to the following tests after 0, 3, 6, and 9 months of aging:

(a) determination of mechanical properties under static tension: tensile strength (R*_m_* [MPa]) and Young’s modulus (E*_t_* [MPa]). Tests were conducted in accordance with the PN-EN ISO 527-1, 2, 4:2012 standard [32] on a multifunctional testing machine from Zwick Roell GmbH & Co. (Ulm, Germany) with the following parameters: tensile speed v = 50 mm/min, measured force range 10 kN.
(b) Charpy impact strength (a*_k_* [kJ m^−2^]) of notched specimens. Tests were conducted in accordance with the PN-EN ISO 179:2001 standard [33] using an electronic Charpy impact tester from VEB Werkstoffprüfmaschine (Leipzig, Germany). A pendulum with a nominal energy of 7.5 J was used for the tests.
(c) rheological: determination of the viscosity average molecular weight using an Ubbelohde viscometer [34,35]. The tests were conducted on samples weighing between 0.2475 and 0.2525 g, which were kept in a water bath at a temperature of 30–40 °C for 20–30 min.

The limiting viscosity (intrinsic viscosity) was calculated based on the Salomon-Ciuta Equation (1) [34,36,37]:

(1) $$\left[\eta \right]=\frac{\sqrt{2}}{c}\sqrt{\eta_{spec}-\ln\eta_{formula}},$$

where

[*η*]—limiting viscosity number [dl g^−1^], c—polymer concentration in the solvent [g dl^−1^], η_formula_—reference viscosity (η_formula_ = t/t_0_), t—flow time of the tested solution [s], t_0_—pure solvent flow time [s], η_spec_—specific viscosity (η_spec_ = η_formula_ − 1).

The viscosity average molecular weight was determined using the Mark–Houvink equation [35] for low-concentration Equation (2):

(2) $$\left[\eta \right]=K{M_{v}}^{\alpha },$$

where

[η]—limiting viscosity number [dL g^−1^], K,α—constants determined experimentally for individual polymers in specific solvents and temperatures, M_v_—molecular weight.

After transforming the relationship, we can calculate the viscosity-average molecular weight using Equation (3):

(3) $$M_{v}=\sqrt[{\alpha }]{\frac{\left[\eta \right]}{K}}$$

Chloroform was used as the solvent for polylactide and WPC.

For the calculation of the viscosity-average molecular weight (M_v_), the same Mark–Houwink parameters (K = 2.28 × 10^−4^ dL/g, α = 0.76) were applied for both PLA and WPC samples, as literature reports [27] indicate that in chloroform the viscometric response is governed by the soluble PLA matrix, while the lignocellulosic filler remains insoluble and does not significantly influence the hydrodynamic volume of the polymer chains.

Mechanical property tests were performed in five replicates for each sample, resulting in a total of 40 samples. Rheological tests were performed in three replicates for each sample and test period, resulting in a total of 24 samples.

## 3. Results and Discussion

**Figure 2:**
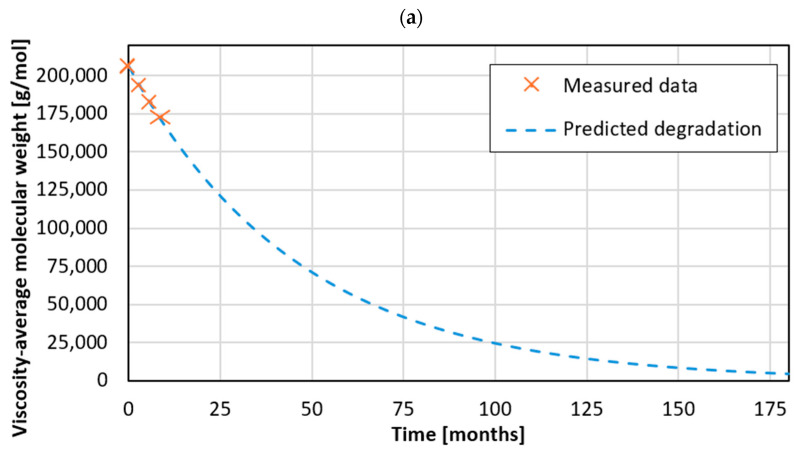

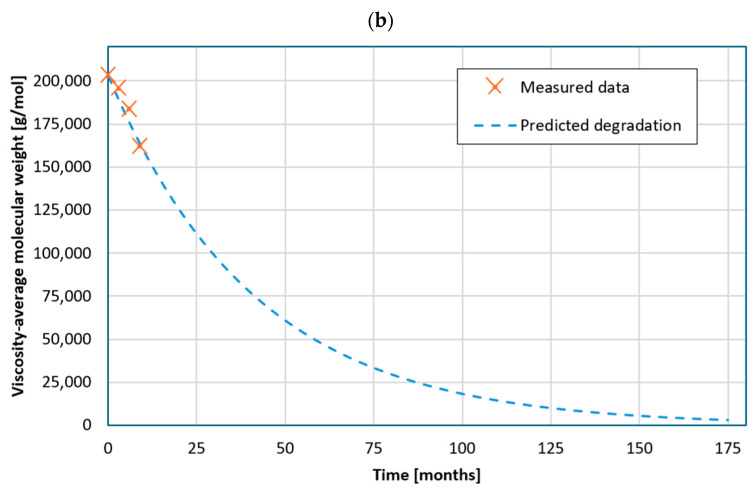
Change in the viscosity-average molecular weight for samples made of: (**a**) rPLA, (**b**) WPC, after aging along with predicted degradation over time.

Figure 2a,b show the change in the viscosity average molecular weight (M_v_) as a function of aging time for samples made of recycled polylactide (Figure 2a) and wood–polymer composite (Figure 2b).

Analyzing Figure 2a, a decreasing trend in the molar mass (M*_v_*) was observed for samples made of rPLA, which may indicate gradual degradation of the material in a seawater environment. The initial M*_v_* value of the samples was 205.907 g mol^−1^, while after 3 months of aging it decreased to 193.552 g mol^−1^, representing a decrease of approximately 6%. After 6 months, the M*_v_* value reached 182.595 g mol^−1^, representing a decrease of approximately 11% compared to the initial value. However, after 9 months of aging, the molar mass of the rPLA samples decreased to 172.812 g mol^−1^, which corresponds to a decrease of 16% compared to the initial value. On average, the viscosity-average molecular weight in seawater (i.e., the aggressive environment) decreased by 11%. Based on the obtained results, it was concluded that the reduction in molar mass is not linear, and the rate of this process gradually decreases. The largest decrease was observed in the first time interval (0–3 months), when the molar mass decreased by 12.355 g mol^−1^. In subsequent periods, decreases of 10.957 g mol^−1^ in 3–6 months and 9.783 g mol^−1^ in 6–9 months were observed, respectively, suggesting a slowdown in the degradation process, likely due to the deposition of microorganisms on the sample surface or a reduction in the availability of degradation factors. The degradation rate constant k for the given data in the second-order model is 0.0213 [1 g·mol·month^−1^].

These observations suggest that in the case of rPLA, the degradation process is primarily hydrolytic and proceeds rapidly in the initial stage due to the diffusion of water molecules into the amorphous regions of the polymer. Over time, the process slows down, which may be related to the formation of a surface biofilm layer composed of adhered microorganisms. This layer limits the diffusion of water and oxygen into the inner regions of the material, passivating the surface and reducing the rate of hydrolysis. Such behavior has been previously described for PLA-based materials by Emadian et al. [27] and Tokiwa et al. [38], who reported that biofilm formation decreases the rate of ester bond cleavage during long-term exposure in aquatic environments.

In the next part of the analysis, the effect of exposure to seawater on the properties of the WPC sample was examined. A decreasing M*_v_* value was observed (Figure 2b), similar to the phenomenon observed in the rPLA sample, indicating gradual degradation of the material in marine conditions. The initial M*_v_* value was 202.702 g mol^−1^, but after 3 months it decreased to 195,688 g mol^−1^, representing a decrease of approximately 4%. After 6 months, the M*_v_* value reached 183.844 g mol^−1^, representing a 9% decrease from the initial value. After 9 months, the molar mass decreased to 161.952 g mol^−1^, corresponding to a decrease of approximately 20% from the initial value. On average, the viscosity-average molecular weight decreased by 10% during aging in seawater (i.e., an aggressive environment). Based on the obtained results, it was found that the reduction in molar mass, similarly to the previous materials, does not occur linearly. However, the rate of this process in the case of the WPC gradually accelerates rather than slows down [39]. The smallest decrease was observed in the first time interval (0–3 months), when the molar mass decreased by 7014 g mol^−1^. In subsequent periods, a greater decrease in M*_v_* values was observed: by 11,844 g mol^−1^ (3–6 months) and 21,892 g mol^−1^ (6–9 months), respectively. These results suggest that degradation intensifies over time, which may be the result of a gradual weakening of the material structure or the increasing influence of external factors such as hydrolysis or environmental factors. The degradation rate constant k for the given data in the second-order model is 1.085 × 10^−7^ [1 g·mol·month^−1^].

The progressive increase in degradation rate observed for the WPC indicates a more complex degradation mechanism involving both hydrolysis of the PLA matrix and biological decomposition of the lignocellulosic filler. The presence of wood flour facilitates water absorption and increases porosity, allowing more effective penetration of seawater and microorganisms into the composite structure. Cellulolytic microorganisms can decompose the wood components, causing local loss of interfacial adhesion between the polymer matrix and the filler, which promotes microcrack formation and accelerates further hydrolysis of the PLA phase. This mechanism is consistent with literature reports on PLA/wood composites exposed to marine and humid environments [25]. Therefore, the degradation of WPC in seawater can be described as a hydro-biodegradation process, where the biological decay of the lignocellulosic phase enhances the overall rate of molecular chain scission.

Although the total reduction in molecular weight after 9 months was higher for WPC (20%) than for rPLA (16%), this difference does not indicate a faster degradation rate of the WPC during the entire exposure period. Kinetic analysis indicated that rPLA experienced a higher initial degradation rate, particularly within the first 0–3 months of exposure, as confirmed by the elevated degradation rate constant (k = 0.0213 [1 g·mol·month^−1^]) compared to WPC (k = 1.085 × 10^−7^ [1 g·mol·month^−1^]). This means that rPLA undergoes rapid hydrolytic chain scission at the early stage of immersion, whereas in the WPC the process gradually accelerates due to the progressive biological decomposition of the lignocellulosic filler, which increases porosity and facilitates water penetration. Therefore, rPLA degrades faster initially, while the overall degree of degradation after prolonged exposure is greater for WPC due to the combined effect of hydrolysis and biodegradation.

Comparative analysis of all tested samples showed that the second-order kinetic model accurately reflects the degradation process of waste polylactide (Figure 2a). The reduction in viscosity-average molecular weight with time occurs nonlinearly, which is characteristic of degradation mechanisms leading to the gradual cleavage of bonds in the macromolecular structure. The rate of M*_v_* decrease for rPLA decreases with time, meaning that degradation initially occurs faster and then slows down. However, in the case of the WPC (Figure 2b), the rate of M*_v_* decrease accelerated with time.

The alterations in the mechanical properties of the examined materials are directly correlated with the observed reduction in M*_v_*, indicating a degradation of the polymer’s structural integrity as a consequence of seawater-induced aging [39].

Analysis of M*_v_* variations indicates an approximate 16% decrease over a 9-month interval, aligning with the degradation mechanisms of PLA documented in the literature [26].

**Figure 3:**
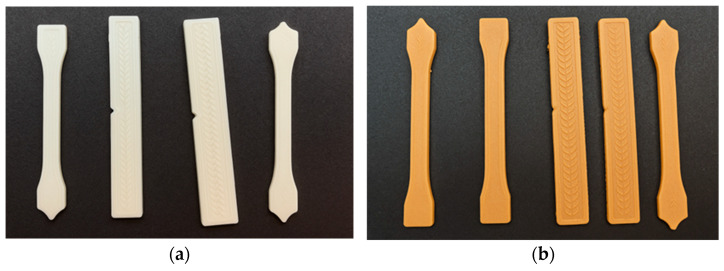
View test specimen: (**a**) rPLA, (**b**) WPC.

Image analysis of samples after a 9-month aging process (Figure 3a,b) revealed no significant changes in the surface morphological properties compared to the reference samples (Figure 1a,b).

Only slight clouding and warping were observed compared to the samples before the aging process (Figure 1a,b) in the seawater environment. Samples printed using the FDM technique may have internal residual stresses, which, upon degradation, can lead to deformation.

**Figure 4:**
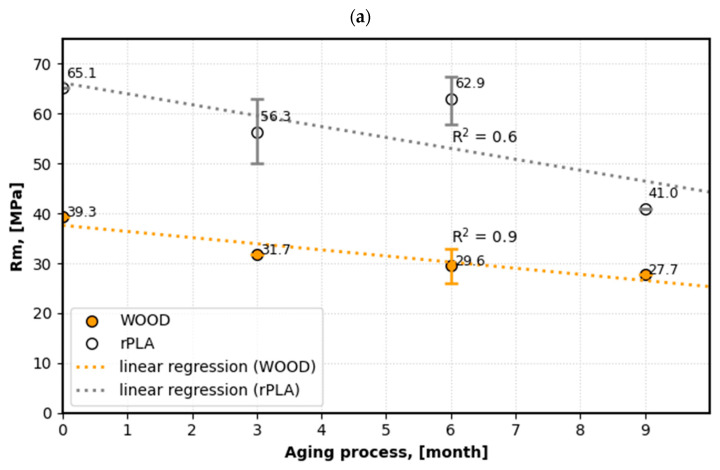

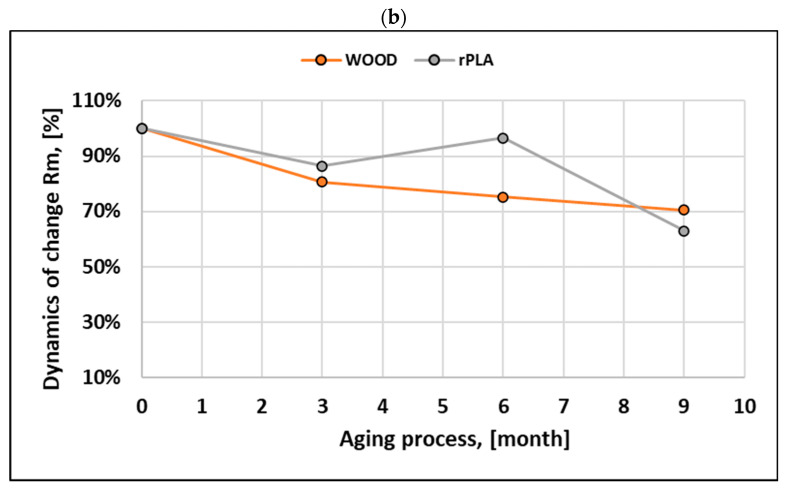
The influence of the aging process on the change in the value of mechanical properties under static tension: (**a**) tensile strength—R*_m_* [MPa], (**b**) dynamics of changes in tensile strength—R*_m_*.

**Figure 5:**
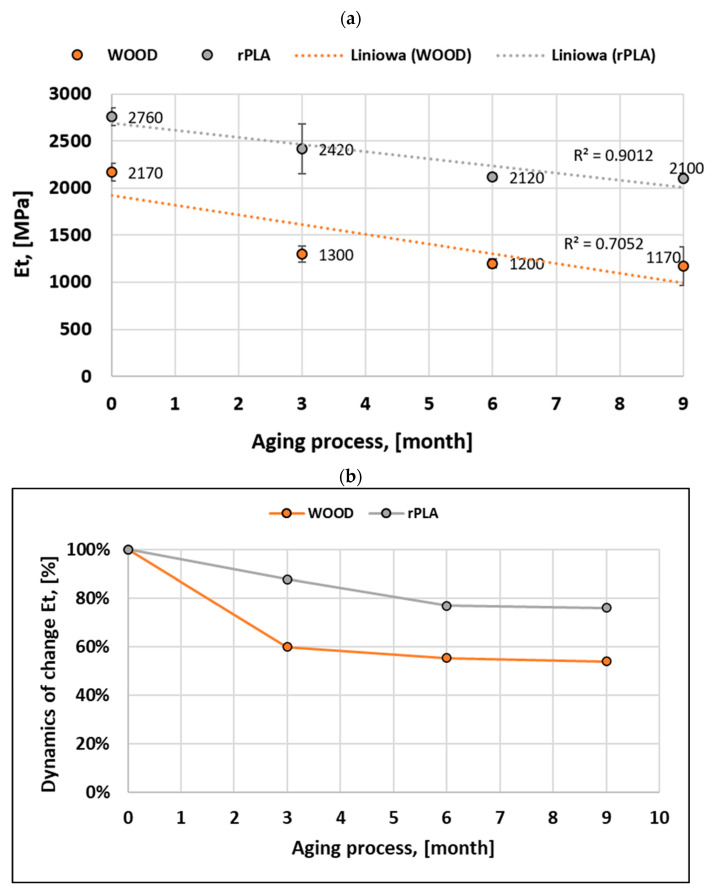
The influence of the aging process on the change in the value of mechanical properties under static tension: (**a**) Young’s modulus—E_t_ [MPa], (**b**) dynamics of changes in Young’s modulus—E_t_.

**Figure 6:**
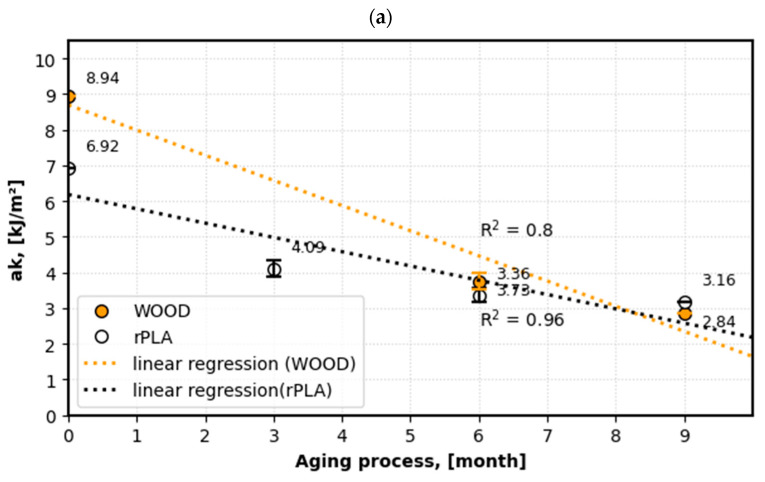

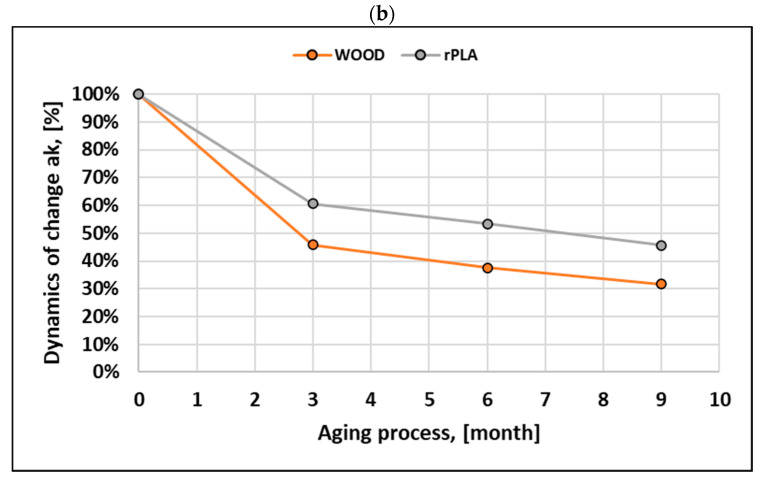
The influence of the aging process on the change in the value of mechanical properties: (**a**) impact strength ak [kJ/m^2^], (**b**) dynamics of change.

Figure 4, Figure 5 and Figure 6 present the effect of aging on the mechanical properties of additive manufacturing products.

An analysis of the impact of nine months of exposure to a seawater environment on tensile strength (Figure 4a) revealed a reduction in the measured values across all tested materials, including rPLA and WPC (wood polymer composite: PLA + MD).In the case of recycled polylactide (rPLA)—Figure 4a, a decreasing trend in the R*_m_* value was observed, which indicates gradual degradation of the material in the seawater environment. The initial R*_m_* value was 65.1 MPa, while after 3 months of aging it decreased to 56.3 MPa, which represents a decrease of approximately 14% compared to the initial value, i.e., the samples before the aging process. After 6 months, the Rm value reached 62.9 MPa, which represents a decrease of approximately 3% compared to the initial value. However, after 9 months of aging, the R*_m_* of the rPLA samples decreased to 41.0 MPa, corresponding to a decrease of approximately 37% compared to the initial value. On average, the tensile strength of the sample stored in the seawater environment (i.e., aggressive environment) decreased by approximately 18%. The greatest decrease was recorded in the third time interval (6–9 months), when the Rm value decreased by 34%. This suggests a rapid acceleration of the degradation process, likely due to changes in the polymer structure associated with the breakage of polymer chains.

In the case of WPC samples, a decreasing trend in the R*_m_* value was observed throughout the storage period, with stable dynamics of property changes (Figure 4b), which indicates gradual degradation of the material in a seawater environment. The initial Rm value was 39.3 MPa, while after 3 months of aging it decreased to 31.7 MPa, representing a decrease of approximately 19% compared to the initial value. After 6 months, the Rm value reached 29.6 MPa, representing a decrease of approximately 24% compared to the initial value. After 9 months of aging, the R*_m_* of WPC samples decreased to 27.7 MPa, corresponding to a decrease of approximately 30% compared to the initial value. On average, the tensile strength of the sample stored in the seawater environment decreased by approximately 24%. The greatest decrease in value was recorded in the first time interval of the aging process (0–3 months), where the Rm value decreased by 19%.

In the case of WPC samples, the dynamics of property decline is uniform, as shown in Figure 4b (orange graph), suggesting that the degradation process proceeds at a similar, relatively constant rate in each of the analyzed periods.

Analyzing the change in the elastic modulus E_t_ (Figure 5a,b) of polymeric materials during the aging process in a seawater environment, a decrease in the tested value was observed for all materials. In the case of recycled polylactide (rPLA), a decreasing trend in the Et value was observed, which, similarly to the Rm value, may indicate gradual degradation of the material in the seawater environment. The initial Et value was 2760 MPa, while after 3 months of aging it decreased to 2420 MPa, representing a decrease of approximately 12% compared to the initial value, i.e., the samples before the aging process. After 6 months, the Et value reached 2120 MPa, representing a decrease of approximately 23% compared to the initial value. However, after 9 months of aging, the Et of recycled polylactide decreased to 2100 MPa, corresponding to a decrease of approximately 24% compared to the initial value. On average, the elastic modulus of the sample stored in a seawater environment (i.e., an aggressive environment) decreased by approximately 20%. The largest decrease in properties was observed in the initial phase of the aging process, i.e., in the period from 0 to 3 months, where the value decreased by approximately 12%. In subsequent periods, a similar decrease in properties was observed compared to the first period, until degradation ceased in the third storage period, i.e., from 6 to 9 months (approximately 1% change). These observations were confirmed by analysis of the dynamics of the aging process for the rPLA material (Figure 5b—gray line).

In the case of WPC samples, a decreasing trend in the Et value was also observed throughout the storage period, which indicates gradual degradation of the material in the seawater environment. The initial Et value was 2170 MPa, while after 3 months of aging it decreased to 1300 MPa, which represents a decrease of approximately 43% compared to the initial value. After 6 months, the Et value reached 1200 MPa, representing a decrease of approximately 48% compared to the initial value. After 9 months of aging, the Et value of the WPC samples decreased to 1170 MPa, representing a decrease of approximately 51% compared to the initial value. On average, the Young’s modulus of the sample stored in the seawater environment decreased by approximately 47%. The largest and most rapid decrease was observed in the first time interval of the aging process (0–3 months), where the Et value decreased by 43%. In subsequent phases, a slowdown in the material degradation process was observed, and its properties stabilized, as confirmed by the analysis of the dynamics of changes in the material’s parameters, presented in Figure 5b (orange line).

In the next stage, the effect of the seawater environment on the impact strength of the tested materials was analyzed (Figure 6a,b). For both materials, a decreasing trend was observed (Figure 6a), with similar dynamics of change during the studied periods (Figure 6b).

In the first time interval of the aging process (0–3 months), the most intense and rapid reduction in the ak coefficient was observed, reaching approximately 39% for rPLA and approximately 54% for WPC. In subsequent stages of the degradation process, both rPLA and WPC exhibit a slower rate of property loss, leading to stabilization of material parameters. This phenomenon was confirmed by the analysis of the dynamics of property changes, presented in Figure 6b.

Analysis of the results suggests that both rPLA and WPC do not exhibit complete resistance to prolonged exposure to aquatic environments. The hydrolysis process leads to more rapid degradation of rPLA, whereas WPC degrades at a slower rate, primarily due to the breakdown of the organic components within the materials. The rate of degradation directly impacts the mechanical properties of the materials, which is crucial when selecting appropriate materials for applications requiring resistance to aggressive conditions, particularly marine environments. The obtained data confirm the need for routine monitoring of changes in average molecular weight (M*_n_*) as a primary indicator of deterioration in polymers and composites during the aging process.

## 4. Conclusions

Based on the conducted research the following conclusions can be drawn:

1. The observed degradation behavior of recycled polylactide (rPLA) and the wood–polymer composite (WPC) confirms that the rate and mechanism of polymer deterioration in marine environments are strongly dependent on the material’s molecular architecture and the nature of its constituents. Hydrolytic scission of ester bonds in rPLA occurs readily under marine exposure, while the presence of lignocellulosic fillers in WPC promotes additional biodegradation pathways associated with microbial and enzymatic activity.
2. The correlation between the reduction in molecular weight and the decline in mechanical performance demonstrates that the macromolecular degradation of polymer chains directly translates into the loss of load-bearing capacity. Molecular weight (M_v_) can therefore serve as a reliable quantitative indicator of structural integrity loss and a diagnostic parameter for assessing long-term material performance in marine conditions.
3. Wood-based polymer composites exhibit a complex degradation profile resulting from the interplay of hydrolytic, microbial, and mechanical factors. Although the organic phase accelerates biodegradation, it also contributes to partial stabilization of the composite’s mechanical performance in the early exposure stages due to its ability to absorb and redistribute stresses.
4. The findings emphasize that both recycled PLA and wood-reinforced PLA composites require further optimization to balance mechanical durability and environmental degradability. Material design strategies should focus on controlled hydrolysis kinetics, improved interfacial adhesion between polymer and filler, and the use of surface modifiers or coupling agents that limit water diffusion while maintaining biodegradability.

Future research should aim at systematically quantifying the influence of temperature, salinity, and biological activity on the degradation kinetics of additively manufactured biopolymers. Expanding the study to include diverse filler types and concentrations will allow the identification of compositional thresholds that ensure sufficient operational stability under marine exposure. Moreover, assessing the chemical nature and ecotoxicity of degradation products will be essential for evaluating the environmental compatibility of biodegradable composites and guiding the development of next-generation materials with predictable life cycles and reduced ecological impact.

## Figures and Tables

**Table 1:** Filament properties: rPLA, WPC (PLA + MD) (supplier’s safety data sheet—in the order Fiberlogy S.A., Spectrum).

Properties	Units	Type of Material		Standard
		rPLA	WPC (PLA + MD)	
Density, ρ	g cm^−3^	1.2–1.3	1.19	ISO 1183 [31]
Tensile strength, R_m_	MPa	53.5	53	ISO 527 [32]
Elongation at break, ε	%	5.5	ND	ISO 527 [32]
Elastic modulus, E_t_	MPa	3420	3500	ISO 527 [32]
Charpy impact strength, a_k_	kJ m^−2^	7.5	16	ISO 179 [33]
Filament diameter	mm	1.75 ± 0.05		-

**Table 2:** Parameter summary of additive manufacturing parameters in FDM technology (own study).

Parameters	Units	Value
Initial printing temperature	°C	230
Printing temperature	°C	200
Temperature plate	°C	60
Print speed	mm s^−1^	50
Infill speed	mm s^−1^	50
Wall speed	mm s^−1^	25
Diameter nozzle	mm	1.75

## Data Availability

The original contributions presented in this study are included in the article. Further inquiries can be directed to the corresponding author.

## Abbreviations

ABS	Acrylonitrile Butadiene Styrene
ak	Charpy impact strength
BD	No data (used in tables)
c	Polymer concentration in solvent
Et	Young’s modulus
FDM	Fused Deposition Modeling
ISO	International Organization for Standardization
J	Joule (unit of energy)
k	Degradation rate constant
K, α	Mark–Houwink constants
MD	Wood flour (component of PLA + MD composite)
M*_n_*	Number-average molecular weight
M*_v_*	Viscosity-average molecular weight
PETG	Polyethylene Terephthalate Glycol
PLA	Polylactic Acid
PN-EN	Polish Standard—European Norm
PP	Polypropylene
rPLA	Recycled Polylactide
R*_m_*	Tensile strength
t	Flow time of polymer solution
t_0_	Flow time of pure solvent
UV	Ultraviolet
WPC	Wood–polymer Composite
[η]	Intrinsic viscosity
η_formula_	Relative viscosity
η_spec_	Specific viscosity
